# State of the Art of Interpersonal Physiology in Psychotherapy: A Systematic Review

**DOI:** 10.3389/fpsyg.2017.02053

**Published:** 2017-11-24

**Authors:** Johann R. Kleinbub

**Affiliations:** Department of Philosophy, Sociology, Education and Applied Psychology, University of Padova, Padua, Italy

**Keywords:** psychotherapy, counseling, interpersonal physiology, physiologic concordance, physiological linkage, physiological synchrony, physiological synchronization, psychophysiology

## Abstract

**Introduction:** The fast expanding field of Interpersonal Physiology (IP) focuses on the study of co-ordination or synchronization dynamics between the physiological activities of two, or more, individuals. IP has been associated with various relational features (e.g., empathy, attachment security, rapport, closeness…) that overlap with desirable characteristics of clinical relationships, suggesting that the relevant studies might provide objective, economical, and theory-free techniques to investigate the clinical process. The goal of the present work is to systematically retrieve and review the literature on IP in the field of psychotherapy and psychological intervention, in order to consolidate the knowledge of this research domain, highlight its critical issues, and delineate possible developments.

**Method:** Following the guidelines by Okoli and Schabram (2010), a systematic literature search was performed in Scopus, Web of Science, PsycINFO, and PubMed databases by means of multiple keyword combinations; the results were integrated with references to the retrieved articles' bibliography as well as to other published reviews on IP.

**Results:** All the retrieved documents reported clinical interactions that are characterized, at least partially, by IP phenomena. They appear to use fragmented and sometimes ambiguous terminology and show a lack of both specific theory-informed hypotheses and sound analytical procedures.

**Conclusion:** Although the psychological nature of IP and its role in the clinical relationship are still mostly unknown, the potential value of a physiology-based measure of implicit exchanges in psychotherapy drives an acceleration in this research field. On the basis of the highlighted critical issues, possible future directions for clinical IP researchers are discussed.

## Introduction

There is growing interest in the study of co-regulation of nonverbal behavior and physiological activations in interpersonal contexts. In the last few years various reviews on the topic have been published (Riess, 2011; Delaherche et al., 2012; Butler and Randall, 2013; Koole and Tschacher, 2016; Palumbo et al., 2016; Finset and Ørnes, 2017), vouching for the existence of a general phenomenon of dynamic regulation between interacting people, which is expressed through a wide range of modalities (*body movement, facial expressions, eye gaze direction, face blushing, pupil dilation, skin conductance activity, heart rate variability, breathing rate, paraverbal behaviors, language style*, and more), and most often described in loosely defined terms of synchrony. Among the many modalities through which these coordination phenomena are expressed, the physiological ones, under the umbrella term of interpersonal physiology (IP), are of special interest in the clinical field, as: (a) they are not directly observable by the clinician or a trained observer and therefore promise an additional layer of information on the clinical process; (b) controlling them voluntarily requires significant effort, and generally is much more difficult in comparison to behavioral forms of synchronization; (c) in most cases, they are outside of the individuals' awareness.

While a consensus on the core mechanisms and on the meaning of this phenomenon is still lacking, IP phenomena have been associated with various psychosocial constructs, such as *empathy* (e.g., Marci et al., 2007), *stress contagion* (e.g., Waters et al., 2014), *attachment security* (e.g., Diamond et al., 2008), *marital conflict* (e.g., Levenson and Gottman, 1983), and *emotional regulation* (e.g., Field, 2012), and have been proposed as indexes of relational efficacy in various contexts, such as *teamwork* (e.g., Chanel et al., 2012), *couples* (e.g., Helm et al., 2014), and *psychotherapy/counseling* (e.g., Marci and Riess, 2005).

These characteristics highlight the clear interest that such phenomena represent in the field of psychotherapy research, the focus of which is slowly shifting from the efficacy of approach-specific interventions to the study of relational variables, such as *empathy, alliance, mutual affective regulation*, and, more generally, common factors and micro-processes (e.g., Messer and Wampold, 2002; Orlinsky et al., 2004). Indeed, while research on psychotherapy has widely demonstrated the average efficacy and effectiveness of psychological treatments, the specific factors that drive and inhibit individual change are still not well understood (Lambert, 2013). Whilst research on IP is still in an early phase, it could potentially lead to the development of a tool able to detect the moment-to-moment implicit adjustments that occur between patient and therapist. Such an achievement would represent a major paradigm advancement for research on the clinical process, offering a new set of objective and (in most cases) automatic measurements.

The goal of the present work is to systematically retrieve and review the literature on IP in the field of psychotherapy and psychological intervention, in order to consolidate the knowledge of this research domain, highlight its critical issues, and delineate possible developments.

## Method

Following the guidelines by Okoli and Schabram (2010), a systematic literature review was performed with the purpose of identifying the literature on IP phenomena in a clinical context, analyzing its characteristics, and highlighting both its strengths and its weaknesses. Given the great variety of terms employed to describe IP, three sets of keywords were chosen to identify the pertinent papers, based on the general reviews: a first set assessing the subject of synchronization (synchron^*^, concordance, attunement, linkage, interpersonal physiol^*^, interpersonal autonom^*^, mimic^*^, mirror^*^, entrainment), a second set specifying the physiological nature (physiol^*^, psychophysiol^*^, neurophysiol^*^, autonom^*^, sympath^*^, parasympath^*^, heart, skin conductance, galvanic skin, gsr, hrv, eeg, ecg, rsa, electroenceph^*^, electromyo^*^, electrocardio^*^, pupil^*^, blush^*^), and a third set specifying the clinical context (psychotherap^*^, rapport, alliance, clinical relation^*^, therapeutic relation^*^, alliance, client, counsel^*^). A wildcard symbol (^*^) was employed to generalize those keywords typically characterized by varying suffixes (e.g., one paper might exclusively employ one of the forms “physiology,” “physiologic,” or “physiological,” the wildcard form “physiol^*^” would match them all) but not for acronyms and words that are most commonly employed in a single form (e.g., “alliance”). The search was performed by fixing a logical conjunction (AND) relationship between the three sets, this means that each result was required to have at least one member of each set. Search areas included the “title,” “abstract,” and “keywords” fields through the following databases: Scopus, Web of Science (core collection), PsycINFO (EBSCO), PubMed. The search results were individually inspected, and only original research articles and case reports, written in English and published in international peer-reviewed journals, were considered. Furthermore, to be considered a match, the studies had to explicitly focus on simultaneous physiological activation of persons involved in a therapeutic or otherwise clinical interaction.

A second in-depth research step was performed by inspecting under the same criteria the works referenced in the articles that were found both in the database search, and in the existing reviews on interpersonal co-regulation (Riess, 2011; Delaherche et al., 2012; Butler and Randall, 2013; Koole and Tschacher, 2016; Palumbo et al., 2016; Finset and Ørnes, 2017).

All the matching articles were retained irrespective of their methodological quality or year of publication. From each retrieved article, the following information was extracted: (a) the terms employed to define IP; (b) the theoretical framework employed to explain IP (c) the physiological measurements employed; (d) the clinical sample and/or the general study design; (e) the specific psychological or clinical constructs hypothesized for their connection to IP; (f) the methodology through which IP was assessed; (g) the general findings.

## Results

As of September 2017, the keyword-based search returned the following number of documents: Scopus = 500, Web of Science = 98, PsycINFO = 163, PubMed = 98. Among these, 10 articles were matching the inclusion criteria. Following the in-depth research step, 9 additional studies were identified; all the 19 included studies are shown in Table 1.

**Table 1:** Review results sorted by year of publication.

**References**	**Terms used**	**Theoretical framework**	**Physiological measures**	**Setting**	**Clinical/psychological construct**	**Interpersonal physiology measure**	**Results**
Di Mascio et al., 1955	“Sociophysiology” “Concordance” “Discordance”	Unspecified	HR	3 dyads, unspecified sessions: individual psychoanalytic psychotherapy	n/a	Correlation	Two examples, of HR “concordance” and “discordance,” were reported.
Coleman et al., 1956	“Physiological relationship”	Counter-transference, empathy (Reich, 1951)	HR	1 dyad, 44 sessions: individual psychoanalytic psychotherapy	Observer-rated patient's emotions (Custom categories: “Anxiety,” “Depression,” “Intra-punitive hostility,” “extra-punitive hostility”)	Graphical comparison of patient's and therapist's HR averaged within each emotion	Patient's and therapist's HR was similar for all emotions except “extra-punitive hostility” characterized by relatively higher therapist's HR.
				Subset of 17 sessions with both “Depression,” and “extra-punitive hostility” emotions	Therapist rated distractions or disturbing thoughts	Similarity of patient's and therapist's HR within the two emotions	Therapist distractions or disturbing thoughts were more frequent in low HR similarity sessions.
Dimascio et al., 1957	“Interpersonal physiology”	Unspecified	HRV	1 dyad, 12 sessions: individual psychoanalytic psychotherapy	Observer-rated salient segments of therapy: Bales Interaction Coding System (BICS; (Bales, 1951); Categories: “Tension,” “Tension release,” “Neutral,” “Disagreement,” “Antagonism”)	Graphical comparison of physiological activations (between-sessions within-subject correlations)	Patient's and therapist's HRV was similar in “Tension,” “Tension release,” and “Neutral” segments, but opposite in “Antagonism” segments.
McCarron and Appel, 1971	n/a	Information theory (Lennard and Bernstein, 1960)	SC	12 dyads, 1 session each: initial interviews; 1 dyad, 12 weekly sessions	Therapists' transcripts analysis with four classes of verbalizations: Reflection, Interpretation, Interrogation, and Confrontation (Pallone and Grande, 1965)	Graphical comparison of regressions results	A correspondence was found between the stimulus specificity of the therapists' verbalizations and the amplitude of patients' and therapists' SC.
Stanek et al., 1973	“Corresponding curve oscillations” “Co-ordination of the curves from doctor and patient”	Unspecified	HR	32 dyads, 1 session each: initial interviews of psychoanalytic treatment with cardiophobic patients	n/a	Graphical comparison of physiological activity in time	Sections of concordant and discordant patterns of HR reactions were reported as related to the content of the conversations.
Robinson et al., 1982	“Matching of phasic responding” “Phasic measure of affect” “Physiological response correlation”	Empathy	ST, SC	21 dyads, 2 sessions each: 30' psychological counseling (only the second session was analyzed)	Patients' perceived empathy: Barrett-Lennard Relationship Inventory's Empathic Understanding Subscale (EUS; Barrett-Lennard, 1986)	Correlations (ST); manual peak matching (SC)	The number of patients' SC peaks matched within 7 seconds with a counselors' peak showed strong correlation with perceived empathy.
Marci and Riess, 2005	“Physiologic concordance”	Empathy	SC	1 dyad, 1 session: individual psychotherapy	n/a	Unspecified time series analysis	A high degree of SC concordance between patient and therapist was reported
Marci and Orr, 2006	“Psychophysiologic concordance”	Empathy	SC	20 dyads, 1 session each: brief semi-structured interview with the same clinician. Half of the interviews were conducted in an emotionally neutral way, while the other half with emotional distance	Interviewer gaze direction; patients' perceived empathy: EUS	Moving window correlations (ratio of positive/negative ones)	SC concordance and perceived empathy were significantly lower in the “emotional distance” dyads.
Marci et al., 2007	“Physiologic concordance”	Empathy	SC	20 dyads, 1 session each: already established psychodynamic psychotherapy	Patients' perceived empathy: EUS; Social-Emotional Ratings: BICS	Moving window correlations (ratio of positive/negative ones)	SC concordance correlated with perceived empathy; during moments of high vs. low SC concordance, there were significantly more positive social- emotional interactions for both patients and therapists.
Stratford et al., 2009, 2012^*^	“Neurophysiological correlates of therapeutic alliance” “Physiological concordance” “Skin conductance resonance” “Therapeutic index”	Alliance	EEG, SC	30 dyads, 6 sessions each: relational client-centered psychotherapy	n/a	Moving window correlations (ratio of positive/negative ones)	Trends of distinct brain areas' activity were observed during high SC concordance, and were observed to vary across sessions.
Kleinbub et al., 2012a,b; Messina et al., 2013^*^	“Physiological concordance”	Empathy	SC	39 dyads, 1 session each. Each of 13 volunteers had a 20-min clinical interview with 3 different interviewers with different levels of training (psychotherapists, psychologists, no training)	Patients' perceived empathy (EUS); Observer rated empathy: Empathic Understanding in Interpersonal Processes Scale (EUIP; Carkhuff, 1969)	Moving window correlations (ratio of positive/negative ones); Lag analysis	SC concordance correlated with perceived empathy but not with observer-rated empathy. The three levels of training predicted higher concordance at different lags.
Seikkula et al., 2015	“Embodied Attunement” “ANS synchrony”	Embodiment, intersubjectivity	SC, HRV	1 tetrad (1 couple and 2 therapists), 1 session: dialogical psychotherapy	n/a	Graphical comparison of physiological activity in time	The clinical micro-process corresponding to dyadic or triadic synchronization of SC and HRV were reported.
Karvonen et al., 2016	“Embodied synchrony” “EDA concordance”	Unspecified	SC	10 tetrads (1 couple and 2 therapists), 1 session each: dialogical psychotherapy	Alliance: Session Rating Scale (SRS; Duncan et al., 2003)	Pairwise moving window correlations (ratio of positive/negative ones); Lag analysis	Higher than random SC synchrony was found in at least one dyad of each tetrad, mostly with a lag up to 1 s. On average therapist-therapist dyads had the most synchrony, while patient-patient ones the least. A correlation between SC synchrony and SRS was reported.
Päivinen et al., 2016	“Sympathetic synchrony” “Physiologic synchrony”	Unspecified	SC	2 tetrads (1 couple and 2 therapists), 1 session each: dialogical psychotherapy	Blaming: qualitative text analysis	Graphical comparison of physiological activity in time	SC activation of each tetrad's participants during blaming episodes were qualitatively reported. In some occasion therapist showed opposite arousal in respect to patients.
Orsucci et al., 2016	“Synchronization”	Dynamic complex system	SC	1 dyad, 1 session: short term cognitive-based psychotherapy	Linguistic prosody	Principal Component Analysis; Markov Transition Matrix; Cross-Recurrence Quantification Analysis	Cluster analysis of SC and prosodic synchrony reveals stable states that may match specific clinical processes. Further nonlinear properties of the session data are reported.
Kykyri et al., 2017	“Embodied attunement”	Embodiment	SC	1 tetrad (1 couple and 2 therapists), 1 session: dialogical psychotherapy	Linguistic prosody	Correlations	Various patterns of SC synchrony were qualitatively reported.
Palmieri et al., in press	“Physiological synchronization”	Attachment; Dyad system model	SC	18 dyads, 1 session each: 20′ clinical interviews. Half of the interviewers received a secure attachment priming	Attachment priming (Mikulincer et al., 2001)	Moving window correlations (ratio of positive/negative ones); Lag analysis	The priming procedure increased SC synchrony at negative lags (i.e., therapist leading) but not overall.

*For each study, only the details, methods, and results directly assessing IP were included in this table, not the full design. SC, skin conductance; ST, skin temperature; HR, heart rate; HRV, heart rate variability; EEG, electroencephalography*.

* *Articles sharing both the same dataset and similar design were collapsed in a single row*.

### Trends

**Figure 1:**
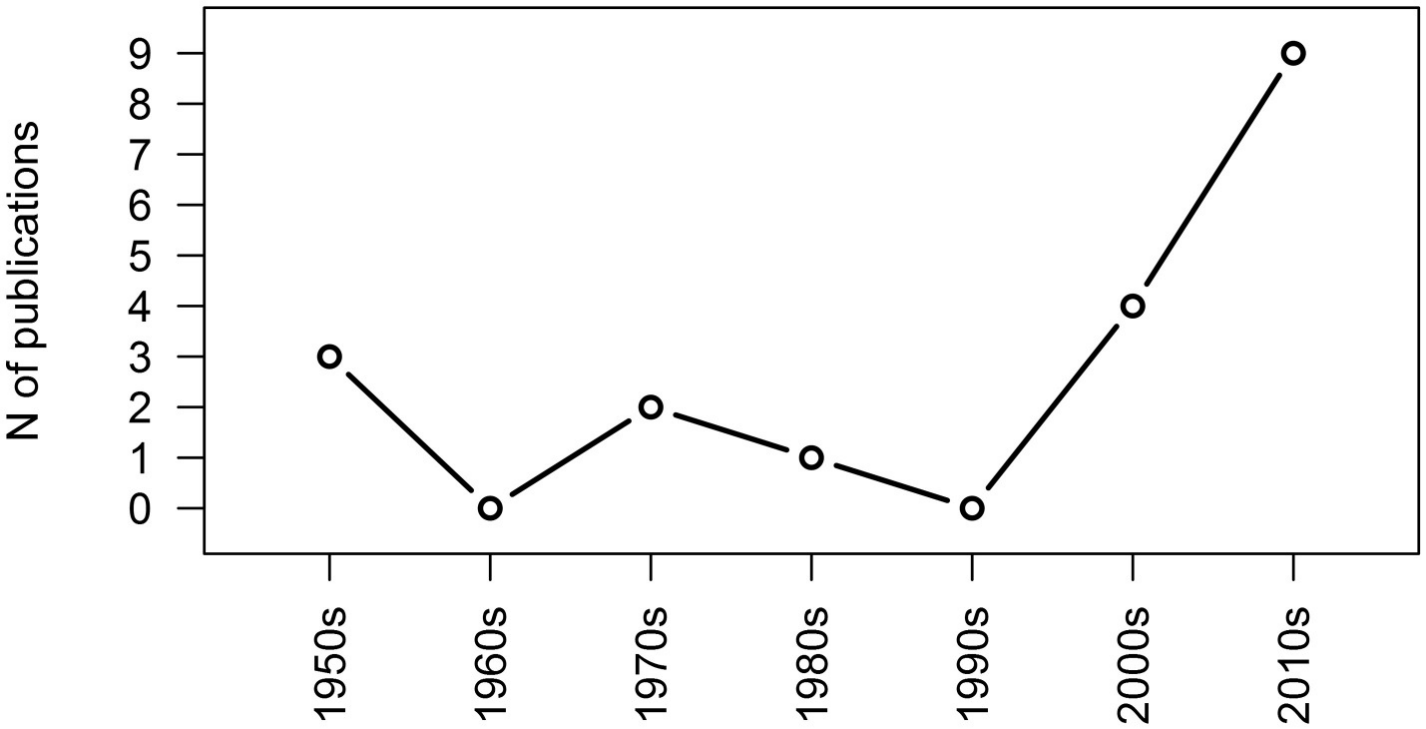
Timeline of publications on IP in psychotherapy by decade.

The first relevant observation on the literature corpus is its time trend. Figure 1 shows, how after an initial exploration of the phenomenon in the second half of the twentieth century, recent years have seen a distinct rediscovery of IP, initiated by the works of Marci and colleagues, that led to an increase in the number of published articles. Indeed, 26% of the whole corpus was published in 2016 or 2017. Yet overall numbers are still small, with only 9 publications since 2010. Research in IP in psychotherapy is still a mostly unexplored niche, lacking consensus in almost every aspect, except the existence of the phenomenon.

### Terminology

Among the many differences, the variety of terms employed to assess the general idea of IP is a big obstacle to the development in the field. It is symptomatic that the broad keyword-based search returned only half of the retrieved articles, and the only recurring terms are *concordance* (*n* = 7) and *synchrony/synchronization* (*n* = 5), combined with a vast number of different descriptors referring to the physiological domain. Indeed, although the overall number of articles retrieved is relatively small, neither of them (nor the previous reviews on the topic, presented in the introduction), managed to report them all.

In order to overcome this difficulty in the future and to aid the establishment of a research field identity, I suggest that further literature follow the advice of the excellent review by Palumbo et al. (2016) to employ *interpersonal physiology* as the most inclusive general domain term.

Even more serious is the fact that the terminological ambiguity extends to the methodologies. While the term *concordance* is mainly associated to the procedure described in Marci and Orr (2006), Marci et al. (2007), the same term has been previously employed to describe simple correlation (Di Mascio et al., 1955), and the same procedure has been also called *physiological synchronizatio*n (Palmieri et al., in press), *skin conductance resonance* (Stratford et al., 2009), *embodied synchrony* (Karvonen et al., 2016), and *therapeutic index* (Stratford et al., 2012). It is imperative that new contributions propose and rely on operationally defined procedures, that explicitly point to the type of IP assessed.

As an example, studies employing the procedure developed by Marci and colleagues, should use *Interpersonal Physiology* in the title, in order to enable quick identification and simpler search, and to explicitly assess which IP measures where employed: *Marci's ratio* or *Marci's index*, in the abstract and methodological sections, instead of vaguely speaking of *synchronization, concordance, attunement*, etc. in the same way authors would specify whether they used a Student's *t*-test or a Mann–Whitney *U*-test procedure instead of referring to a generic comparison of means.

### Theoretical interpretation

Except for McCarron and Appel (1971), none of the studies employed IP as a predictor of a precise theoretical dimension. Five studies did not specify any theoretical interpretation for their data, 2 referred generically to *embodiment theory*, 2 to *system models*, 2 to *alliance*, and most (*n* = 6) to *empathy*, reflecting the general trend of IP literature outside the clinical setting (Palumbo et al., 2016). This lack of specificity is a big obstacle in understanding the clinical meaning of IP. For instance, while its role in the clinical relationships is undisputed, *empathy* is neither a simple nor a single process; instead it is known to consist in the interaction of multiple components, ranging from basic affective processes up to complex cognitive and social dimensions, each of them being characterized by specific neurobiological activations (e.g., Coutinho et al., 2014). Knowing that IP correlates with patients perceived empathy (Marci et al., 2007) has been a great incentive to foster this line of research, but, on the other hand, is a very general, and quite uninformative kind of observation. In order to reach the exciting scientific and clinical achievements promised by IP, we need to start asking more specific questions, such as: *which specific empathic components are responsible for IP? What fundamental intersubjective processes IP does represent?* Furthermore, specifically with regard to the clinical setting, we need to start informing our hypotheses with theoretical constructs of our clinical models (for a broader dissertation on this topic: Salvatore, 2011), for instance: *which constructs from Dynamic Psychology, or Cognitive Behavioral Therapy are associated with IP? Is IP a mechanism involved in interpersonal emotional regulation? In active listening? In transference and counter-transference processes? In projective identification?* And we need to proceed beyond the correlational logic by asking, whether these theoretical constructs map exactly to IP, or only partially, and where eventual discrepancies come from. Such form of reasoning could lead to an evidence-based refinement of the theoretical constructs, but also potentially inform and enrich the ways we observe and measure IP. Indeed, only after the identification of the precise therapeutic dynamics (if any) that IP represents within an established model of psychotherapy, we might be able to employ it to build empirical bridges across models.

Worth of note in this direction is the effort by Ham and Tronick (2009): Converging data from psychotherapy and mother-infant research, the authors propose a theoretical framework, where IP phenomena in therapeutic exchanges can be interpreted in the broader context of attachment theory and dyad models. Although still mostly speculative, the arguments presented in the article are a good and fertile example of how IP research can benefit from, and in return empower, clinical theory.

### Assessment

#### Correlation assumptions

Aside from the studies relying on graphical comparison, and with one exception (Orsucci et al., 2016), the quantitative assessment of IP in this literature relies on correlational methods. Yet there are potential dangers in their blind adoption. Indeed, none of the retrieved studies directly assess the potential violation against methodical assumptions and the associated risks of ending up with spurious correlations. So, for instance, the linearity, and homoscedasticity assumptions of the Pearson correlation in the time series domain translate into the assumption of stationarity. This means not only that the individual signals are assumed to contain no relevant trends or drifts, but also that the linear association among them is constant over time, which, in data collected from human behavior, is almost never the case. Furthermore, if two signals are strongly auto-correlated (i.e., strongly dependent from their previous values), a correlation between them might produce inflated results, even where signals are independent. The most frequently employed physiological signal in the reviewed papers, SC, is indeed highly nonstationary and autocorrelated, and requires either signal processing techniques, such as detrending or deconvolution (e.g., Benedek and Kaernbach, 2010), or specific analytic approaches. The most widely employed strategy in the retrieved literature was the windowing procedure (Boker et al., 2002), a nonparametric approach which substitutes the correlation's assumption of global stationarity with that of local stationarity by calculating correlation over shorter, usually overlapping windows; other authors (Liu et al., 2016) suggested dynamical correlation as a more flexible alternative to the Pearson index. Yet no rigorous comparison and validation of these techniques has been published to date. Furthermore, the correlational approach reflects a general data-driven approach to IP, which has the advantage of providing a very direct and un-mediated assessment of the phenomenon, but, at the same time, the disadvantage of being only a measure of very simple linear association. Other methodologies, e.g., system dynamics (Ferrer and Helm, 2013), might be better suited to model more sophisticated and theoretically informed relationships between the dyad's signals.

#### Lag analysis

Analogously, in the reviewed articles, the analysis of lag and the direction of influence were performed through very simple procedures such as applying a constant lag to the whole time-series and comparing the resulting windowed cross-correlation of various lag settings. This approach has two main limitations: first it does not model the lag fluctuations during the interaction (especially in longer interactions characterized by multiple turn changes, such as a psychotherapy session, the assumption of a constant lag might lead to spurious results); and second, while the lagged-correlation can assess a temporal association between two phenomena (such as SC peaks), the method does not allow to imply causation (i.e., one person's activation causing another's at a later time) and thus it can't be established if a high synchronization at a specific lag is caused by leading-pacing dynamics or just by synchronization at different phases. This latter limitation can be overcome by employing specific causality tests such as Granger causality (Gourévitch et al., 2006; Liu and Molenaar, 2016). Granger causality implies not only a time-lagged association between two signals, but that knowing the previous states of the first signal (the *leading* signal) allows a better prediction of the second signal (the *pacing* signal) than just knowing the previous states of the second signals. Like most parametric analytic tools for time series, Granger causality has strong assumptions, and might return spurious results on nonstationary and cointegrated series, requiring a cautious implementation in IP data. Just as for the cross-correlation approach, Granger causality in nonstationary data can be analyzed by using a windowing technique (Hesse et al., 2003), or through specific solutions (Toda and Yamamoto, 1995). In conclusion, the technique has already been used to assess IP directionality in one publication on choir singers (Müller and Lindenberger, 2011), yet the concrete advantages over the simpler approaches and the overall validity of the procedure in this field are still unclear.

#### Choice of parameters

Another issue concerns the many degrees of freedom that multivariate time-series analysis imposes. In the windowed cross correlation approach, the choice of the windows size and increments, the lag interval size and range and the analytic approach for lag, are fixed parameters, which can be combined in a vast range of possible configurations, possibly altering the results in a radical way. For instance, the results obtained by using 30 s windows and 10 s lags might give very different results than using 10 s windows with 30 s lag. While most papers employing this methodology reported the same settings, originally proposed by Marci and Orr (2006), offering at least the comparability of their results, the authors of that original paper did not provide a rationale backing their choice of parameters. The consequences of this blind adoption can lead to seriously skewed conclusions, and generally hinders the possibility of moving from exploratory to confirmatory designs, as clearly explained in the methodological commentary “*The garden of forking path*” by Gelman and Loken (2013). A new generation of research should establish its procedures on an empirical basis, for instance by selecting parameters that maximize the effect size of IP of real dyads in comparison to random data, and by questioning whether absolutely best parameters for human interaction exist, or context-specific variations are to be employed.

#### Interpretation of negative results

Interpreting the analytic results is a critical issue too. In the correlational approaches, the resulting values for a given period of time can be either positive, zero, or negative; it is not clear, whether the high negative correlations (i.e., toward −1) should be considered as a part of IP just like positive values (an approach followed by the Motion Energy Analysis literature, e.g., Ramseyer and Tschacher, 2011), or as the lack of IP [as in the index described by Marci and Orr (2006), and used in several following studies], or possibly as a different form of co-regulation underlying different behaviors (a yet unexplored direction). These can be important differentiators of measured IP processes, which have mostly been neglected in current publications, and generally with a terminological variety similar to that of the main construct. For instance, among the selected papers only Di Mascio et al. (1955) explicitly acknowledged the phenomenon of an inverse correlation between patients' and therapists' physiology, and labeled it *discordance*, while other authors, outside the clinical field, employed different terms such as *anti-phase physiological linkage* (Reed et al., 2013) or *complementarity* (Dale et al., 2013). The lack of observable IP (i.e., very small or close to zero correlation) has not been explicitly addressed in the reviewed literature, while in other IP publications it has been referred as *asynchrony* (Dale et al., 2013); instead the term *desynchronization*, which is very common in neuroscience (e.g., “alpha desynchronization,” or “circadian system desynchronization”), is not used in IP field.

#### How much synchronization?

Finally, the implicit assumption present in most studies that “more IP” (in whatever way one may choose to measure it) is always better, might be an oversimplification. In the broader literature on interpersonal synchrony, some authors found that high synchrony was not necessarily connected with good interpersonal outcomes (Levenson and Ruef, 1992), and a study on mother-infant dyads found both very high and very low levels of prosodic synchronization to be predictive of insecure attachment (Jaffe et al., 2001).

In conclusion these results suggest that IP in psychotherapy should be studied with a higher degree of sophistication, testing theory-driven hypotheses on what amount of IP is advisable, in what context, if the lack of IP or phase opposition could have clinical meanings, and how to correctly assess and interpret lagged synchrony and causal direction.

### Design

The nature of IP and its role in psychotherapy are still mostly unknown, as its relationship with the main psychological dimensions, or its dependency from the dyad's members individual factors, such as gender, diagnosis, or attachment style (just to name a few). Yet 11 out of 19 studies employed a nomothetic design, averaging across dyads or sessions, and comparing group measures to questionnaire data and other constructs in a confirmative fashion. The results of such an approach are low power studies that are unable to make strong points. Research on IP in psychotherapy, at this stage, could probably benefit more from an idiographic type of design, in which physiological dynamics are used either:

– In comparison to theoretically-informed analysis of the clinical content. Specifically models that describe micro-processes and use empirically sound and manualized content analysis procedures are promising candidates. Examples of those are *ruptures and repairs* (Safran et al., 2011), *innovative moments* (Goncalves et al., 2011), or the *patient attachment coding system* (Talia et al., 2017).
– In data-driven, bottom-up procedures of content classification. Therapy sessions contain infinite amounts of information (verbal content, prosody, behavior, individual physiology, etc.) that can be paralleled to IP data to identify significant clusters and dynamics.

Among the available statistical tools to deal with these huge datasets, the Markovian transition matrix procedure (Orsucci et al., 2016) and the Conditional Inference Tree classification approach (Hothorn et al., 2006), can be suggested.

## Conclusions

IP in psychotherapy is a phenomenon reported by numerous independent scholars, and its existence (at least with regard to SC and cardiac activity) can be considered an established fact. Its dynamics and clinical meaning, however, are still almost completely unknown, although there are strong suggestions that the phenomenon might be related to some primitive form of affective empathy such as *emotional contagion* (Coutinho et al., 2014). As highlighted by the growing number of publications and in most authors' argumentations, there is the perception that this line of investigation holds a great value for the field of psychotherapy research. The automation, objectivity, and ecology of the autonomic measures, and their ability to detect implicit intersubjective dynamics, which mostly occur behind the conscious control of patients and therapists, outline the potentials hidden in IP as a research and clinical tool. Yet, to be able to fully benefit from these qualities, a significant amount of basic, idiographic, and/or bottom-up research must be performed. The literature search identified only 19 articles since the 1950s which assess IP in a clinical context, and most of them lack theoretically founded hypotheses, explicitly defined constructs, and operationally defined and empirically validated procedures. To overcome these difficulties, the research community should strive to converge on common terminologies, to focus on the key questions on the nature and clinical interpretation of IP, and to embrace more sophisticated data analysis approaches. It is going to be a long road, but it will be worth it.

## Author contributions

The author confirms being the sole contributor of this work and approved it for publication.

### Conflict of interest statement

The author declares that the research was conducted in the absence of any commercial or financial relationships that could be construed as a potential conflict of interest.

## References

[B1] Bales R. F. (1951). Interaction Process Analysis: A Method for the Study of Small Groups. Cambridge, MA: Addison-Wesley Press, Inc.

[B2] Barrett-Lennard G. T. (1986). The relationship inventory now: issues and advances in theory, method, and use, in The Psychotherapeutic Process: A Research Handbook, eds Greemberg L. S. Pinsof W. M. (New York, NY: Guilford Press), 439–476.

[B3] Benedek M. Kaernbach C. (2010). A continuous measure of phasic electrodermal activity. J. Neurosci. Methods 190, 80–91. 10.1016/j.jneumeth.2010.04.028 20451556 PMC2892750

[B4] Boker S. M. Rotondo J. L. Xu M. King K. (2002). Windowed cross-correlation and peak picking for the analysis of variability in the association between behavioral time series. Psychol. Methods 7, 338–355. 10.1037/1082-989X.7.3.338 12243305

[B5] Butler E. A. Randall A. K. (2013). Emotional coregulation in close relationships. Emot. Rev. 5, 202–210. 10.1177/1754073912451630

[B6] Carkhuff R. R. (1969). Helping and Human Relations: A Primer for Lay and Professional Helpers. New York, NY: Holt, Rinehart and Winston.

[B7] Chanel G. Kivikangas J. M. Ravaja N. (2012). Physiological compliance for social gaming analysis: cooperative versus competitive play. Interact. Comput. 24, 306–316. 10.1016/j.intcom.2012.04.012

[B8] Coleman R. Greenblatt M. Solomon H. C. (1956). Physiological evidence of rapport during psychotherapeutic interviews. Dis. Nerv. Syst. 17:71. 13305455

[B9] Coutinho J. F. Silva P. O. Decety J. (2014). Neurosciences, empathy, and healthy interpersonal relationships: recent findings and implications for counseling psychology. J. Counsel. Psychol. 61, 541–548. 10.1037/cou0000021 25285714

[B10] Dale R. Fusaroli R. Håkonsson D. D. Healey P. Mønster D. McGraw J. J. (2013). Beyond synchrony: complementarity and asynchrony in joint action, in Paper Presented at the Annual Meeting of the Cognitive Science Society (Berlin).

[B11] Delaherche E. Chetouani M. Mahdhaoui A. Saint-Georges C. Viaux S. Cohen D. (2012). Interpersonal synchrony: a survey of evaluation methods across disciplines. IEEE Trans. Affect. Comput. 3, 349–365. 10.1109/T-AFFC.2012.12

[B12] Diamond L. M. Hicks A. M. Otter-Henderson K. D. (2008). Every time you go away: changes in affect, behavior, and physiology associated with travel-related separations from romantic partners. J. Pers. Soc. Psychol. 95, 385–403. 10.1037/0022-3514.95.2.385 18665709

[B13] Dimascio A. Boyd R. W. Greenblatt M. (1957). Physiological correlates of tension and antagonism during psychotherapy; a study of interpersonal physiology. Psychos. Med. 19, 99–104. 10.1097/00006842-195703000-00002 13420290

[B14] Di Mascio A. Boyd R. W. Greenblatt M. Solomon H. C. (1955). The psychiatric interview: a sociophysiologic study. Dis. Nerv. Syst. 16:4. 13231760

[B15] Duncan B. L. Miller S. D. Sparks J. A. Claud D. A. Reynolds L. R. Brown J. (2003). The Session Rating Scale: preliminary psychometric properties of a “working” alliance measure. J. Brief Ther. 3, 3–12.

[B16] Ferrer E. Helm J. L. (2013). Dynamical systems modeling of physiological coregulation in dyadic interactions. Int. J. Psychophysiol. 88, 296–308. 10.1016/j.ijpsycho.2012.10.013 23107993

[B17] Field T. (2012). Relationships as regulators. Psychology 3, 467–479. 10.4236/psych.2012.36066

[B18] Finset A. Ørnes K. (2017). Empathy in the clinician–patient relationship. J. Patient Exp. 4, 64–68. 10.1177/2374373517699271 28725863 PMC5513642

[B18a] Gelman A. Loken E. (2013). The Garden of Forking Paths: Why Multiple Comparisons Can Be a Problem, Even When There Is No “Fishing Expedition” or “P-hacking” and the Research Hypothesis Was Posited Ahead of Time. Unpublished manuscript, Department of Statistics; Columbia University; Department of Human Development and Family Studies; Penn State University, New York, NY.

[B19] Goncalves M. M. Ribeiro A. P. Mendes I. Matos M. Santos A. (2011). Tracking novelties in psychotherapy process research: the innovative moments coding system. Psychother. Res. 21, 497–509. 10.1080/10503307.2011.560207 21480054

[B20] Gourévitch B. Le Bouquin-Jeannès R. Faucon G. (2006). Linear and nonlinear causality between signals: methods, examples and neurophysiological applications. Biol. Cybernet. 95, 349–369. 10.1007/s00422-006-0098-0 16927098

[B21] Ham J. Tronick E. (2009). Relational psychophysiology: lessons from mother–infant physiology research on dyadically expanded states of consciousness. Psychother. Res. 19, 619–632. 10.1080/10503300802609672 19235090

[B22] Helm J. L. Sbarra D. A. Ferrer E. (2014). Coregulation of respiratory sinus arrhythmia in adult romantic partners. Emotion 14:522. 10.1037/a0035960 24708502

[B23] Hesse W. Möller E. Arnold M. Schack B. (2003). The use of time-variant EEG Granger causality for inspecting directed interdependencies of neural assemblies. J. Neurosci. Methods 124, 27–44. 10.1016/S0165-0270(02)00366-7 12648763

[B24] Hothorn T. Hornik K. Zeileis A. (2006). Unbiased recursive partitioning: a conditional inference framework. J. Comput. Graphical Stat. 15, 651–674. 10.1198/106186006X133933

[B25] Jaffe J. Beebe B. Feldstein S. Crown C. Jasnow M. Rochat P.. (2001). Rhythms of dialogue in infancy: coordinated timing in development. Monogr. Soc. Res. Child Dev. 66, 1–132. 11428150

[B26] Karvonen A. Kykyri V. L. Kaartinen J. Penttonen M. Seikkula J. (2016). Sympathetic nervous system synchrony in couple therapy. J. Mar. Family Ther. 42, 383–395. 10.1111/jmft.12152 26748869

[B27] Kleinbub J. R. Messina I. Bordin D. Voci A. Calvo V. Sambin M. (2012a). Synchronization of skin conductance levels in therapeutic dyads. Int. J. Psychophysiol. 85:383 10.1016/j.ijpsycho.2012.07.055

[B28] Kleinbub J. R. Messina I. Bordin D. Voci A. Calvo V. Sambin M. (2012b). Corrigendum to “Synchronization of skin conductance levels in therapeutic dyads” [Int. J. Psychophysiol. 85 (3) (2012) 383]. Int. J. Psychophysiol. 86:299 10.1016/j.ijpsycho.2012.10.007

[B29] Koole S. L. Tschacher W. (2016). Synchrony in psychotherapy: a review and an integrative framework for the therapeutic alliance. Front. Psychol. 7:862. 10.3389/fpsyg.2016.00862 27378968 PMC4907088

[B30] Kykyri V. Karvonen A. Wahlström J. Kaartinen J. Penttonen M. Seikkula J. (2017). Soft Prosody and embodied attunement in therapeutic interaction: a multimethod case study of a moment of change. J. Construct. Psychol. 30, 211–234. 10.1080/10720537.2016.1183538

[B31] Lambert M. J. (2013). The efficacy and effectiveness of psychotherapy, in Bergin and Garfield's Handbook of Psychotherapy and Behavior Change, 5th Edn., ed Lambert M. J. (New York, NY: Wiley), 169–218.

[B32] Lennard H. Bernstein A. (1960). The Anatomy of Psychotherapy. New York, NY: Columbia University Press.

[B33] Levenson R. W. Gottman J. M. (1983). Marital interaction: physiological linkage and affective exchange. J. Pers. Soc. Psychol. 45, 587–597. 10.1037/0022-3514.45.3.587 6620126

[B34] Levenson R. W. Ruef A. M. (1992). Empathy: a physiological substrate. J. Pers. Soc. Psychol. 63, 234–246. 10.1037/0022-3514.63.2.234 1403614

[B35] Liu S. Molenaar P. (2016). Testing for granger causality in the frequency domain: a phase resampling method. Multiv. Behav. Res. 51, 53–66. 10.1080/00273171.2015.1100528 26881957

[B36] Liu S. Zhou Y. Palumbo R. Wang J.-L. (2016). Dynamical correlation: a new method for quantifying synchrony with multivariate intensive longitudinal data. Psychol. Methods 21, 291–308. 10.1037/met0000071 26867156

[B37] Marci C. D. Ham J. Moran E. Orr S. P. (2007). Physiologic correlates of perceived therapist empathy and social-emotional process during psychotherapy. J. Nerv. Mental Dis. 195, 103–111. 10.1097/01.nmd.0000253731.71025.fc 17299296

[B38] Marci C. D. Orr S. P. (2006). The effect of emotional distance on psychophysiologic concordance and perceived empathy between patient and interviewer. Appl. Psychophysiol. Biofeedback 31, 115–128. 10.1007/s10484-006-9008-4 16724278

[B39] Marci C. Riess H. (2005). The clinical relevance of psychophysiology: support for the psychobiology of empathy and psychodynamic process. Am. J. Psychother. 59, 213–227. 16370130 10.1176/appi.psychotherapy.2005.59.3.213

[B40] McCarron L. T. Appel V. H. (1971). Categories of therapist verbalizations and patient-therapist autonomic response. J. Consult. Clin. Psychol. 37, 123–134. 10.1037/h0031291 5565614

[B41] Messer S. B. Wampold B. E. (2002). Let's face facts: common factors are more potent than specific therapy ingredients. Clin. Psychol. Sci. Pract. 9, 21–25. 10.1093/clipsy.9.1.21

[B42] Messina I. Palmieri A. Sambin M. Kleinbub J. R. Voci A. Calvo V. (2013). Somatic underpinnings of perceived empathy: the importance of psychotherapy training. Psychother. Res. 23, 169–177. 10.1080/10503307.2012.748940 23234457

[B43] Mikulincer M. Gillath O. Halevy V. Avihou N. Avidan S. Eshkoli N. (2001). Attachment theory and reactions to others' needs: evidence that activation of the sense of attachment security promotes empathic responses. J. Pers. Soc. Psychol. 81, 1205–1224. 10.1037/0022-3514.81.6.1205 11761318

[B44] Müller V. Lindenberger U. (2011). Cardiac and respiratory patterns synchronize between persons during choir singing. PLoS ONE 6:e24893. 10.1371/journal.pone.0024893 21957466 PMC3177845

[B45] Okoli C. Schabram K. (2010). A guide to conducting a systematic literature review of information systems research. Work. Papers Inf. Syst. 10, 1–51. 10.2139/ssrn.1954824

[B46] Orlinsky D. E. Ronnestad M. H. Willutzki U. (2004). Fifty years of psychotherapy process-outcome research: continuity and change, in Bergin and Garfield's Handbook of Psychotherapy and Behaviour Change, 5th Edn., ed Lambert M. (New York, NY: Wiley), 307–389.

[B47] Orsucci F. F. Musmeci N. Aas B. Schiepek G. Reda M. A. Canestri L.. (2016). Synchronization analysis of language and physiology in human dyads. Nonlinear Dyn. Psychol. Life Sci. 20, 167–191. 27033132

[B48] Päivinen H. Holma J. Karvonen A. Kykyri V. L. Tsatsishvili V. Kaartinen J. (2016). Affective arousal during blaming in couple therapy: combining analyses of verbal discourse and physiological responses in two case studies. Contemp. Family Ther. 38, 373–384. 10.1007/s10591-016-9393-7

[B49] Pallone N. J. Grande P. P. (1965). Counselor verbal mode, problem relevant communication, and client rapport. J. Counsel. Psychol. 12:359 10.1037/h0022769

[B50] Palmieri A. Kleinbub J. R. Benelli E. Messina I. Sambin M. Voci A. (in press). Attachment security prime effect on skin conductance synchronization in psychotherapists: an empirical study. J. Counsel. Psychol. 10.1037/cou0000273 29494169

[B51] Palumbo R. V. Marraccini M. E. Weyandt L. L. Wilder-Smith O. McGee H. A. Liu S.. (2016). Interpersonal autonomic physiology: a systematic review of the literature. Pers. Soc. Psychol. Rev. 21, 99–141. 10.1177/1088868316628405 26921410

[B52] Ramseyer F. Tschacher W. (2011). Nonverbal synchrony in psychotherapy: coordinated body movement reflects relationship quality and outcome. J. Consult. Clin. Psychol. 79, 284–295. 10.1037/a0023419 21639608

[B53] Reed R. G. Randall A. K. Post J. H. Butler E. A. (2013). Partner influence and in-phase versus anti-phase physiological linkage in romantic couples. Int. J. Psychophysiol. 88, 309–316. 10.1016/j.ijpsycho.2012.08.009 22922526

[B54] Reich A. (1951). On counter-tranference. Int. J. Psychoanal. 32, 25–31.

[B55] Riess H. (2011). Biomarkers in the psychotherapeutic relationship: the role of physiology, neurobiology, and biological correlates of E.M.P.A.T.H.Y. Harv. Rev. Psychiatry 19, 162–174. 10.3109/08941939.2011.581915 21631162

[B56] Robinson J. W. Herman A. Kaplan B. J. (1982). Autonomic responses correlate with counselor-client empathy. J. Counsel. Psychol. 29, 195–198. 10.1037/0022-0167.29.2.195

[B57] Safran J. D. Christopher Muran J. Eubanks-Carter C. (2011). Repairing alliance ruptures. Psychother. Relat. Work Evid. Based Respons. 48, 80–87. 10.1093/acprof:oso/9780199737208.003.0011 21401278

[B58] Salvatore S. (2011). Psychotherapy research needs theory. Outline for an epistemology of the clinical exchange. Integr. Psychol. Behav. Sci. 45, 366–388. 10.1007/s12124-011-9180-9 21785950

[B59] Seikkula J. Karvonen A. Kykyri V. L. Kaartinen J. Penttonen M. (2015). The embodied attunement of therapists and a couple within dialogical psychotherapy: an introduction to the relational mind research project. Family Process 54, 703–715. 10.1111/famp.12152 25810020

[B60] Stanek B. Hahn P. Mayer H. (1973). Biometric findings on cardiac neurosis. III. Changes in ECG and heart rate in cardiophobic patients and their doctor during psychoanalytical initial interviews. Psychother. Psychos. 22, 289–299. 10.1159/000286534 4770539

[B61] Stratford T. Lal S. Meara A. (2009). Neurophysiology of therapeutic alliance. Gestalt J. Aust. N. Z. 5, 19–47. Available online at: http://hdl.handle.net/10453/15953

[B62] Stratford T. Lal S. Meara A. (2012). Neuroanalysis of therapeutic alliance in the symptomatically anxious: the physiological connection revealed between therapist and client. Am. J. Psychother. 66, 1–21. 22523792 10.1176/appi.psychotherapy.2012.66.1.1

[B63] Talia A. Miller-Bottome M. Daniel S. I. (2017). Assessing attachment in psychotherapy: validation of the patient attachment coding system (PACS). Clin. Psychol. Psychother. 24, 149–161. 10.1002/cpp.1990 26596847

[B64] Toda H. Y. Yamamoto T. (1995). Statistical inference in vector autoregressions with possibly integrated processes. J. Econ. 66, 225–250. 10.1016/0304-4076(94)01616-8

[B65] Waters S. F. West T. V. Mendes W. B. (2014). Stress contagion: physiological covariation between mothers and infants. Psychol. Sci. 25, 934–942. 10.1177/0956797613518352 24482403 PMC4073671

